# Nutritional prehabilitation strategies in abdominal surgery

**DOI:** 10.1097/MCO.0000000000001215

**Published:** 2026-02-02

**Authors:** Wayne Fradley, Sawsan Abdul-Hamid, Bethan E. Phillips

**Affiliations:** a Centre of Metabolism, Ageing, & Physiology (CoMAP), School of Medicine, University of Nottingham; b Department of Surgery, Royal Derby Hospital, Derby, UK

**Keywords:** abdominal surgery, malnutrition, nutritional assessment, nutritional intervention, prehabilitation

## Abstract

**Purpose of review:**

Many patients undergoing abdominal surgery are considered at-risk of malnutrition and may have a multitude of modifiable risk factors for adverse surgical outcomes. Prior to surgery, risk factors should be identified and mitigated via prehabilitation. This review aims to highlight recent research in nutritional screening, assessment and interventions being incorporated into surgical prehabilitation programmes.

**Recent findings:**

Nutritional screening identifies at-risk patients most likely to benefit from prehabilitation. Assessment of body composition using radiological methods provides an integrated accurate means of risk stratification, allowing intervention in those most likely to benefit. Biochemical immune-nutrition prognostic markers may provide a useful adjunct but lack robust clinical evidence. Unimodal nutritional prehabilitation interventions have mixed evidence of benefit in improving clinical outcomes, such as infectious complications and length of stay. Multimodal interventions are considered more pragmatic and may positively impact functional outcomes and reduce complication rates.

**Summary:**

Utilizing nutrition as part of multimodal prehabilitation shows promise for improving clinical and functional outcomes yet requires strong collaboration between key stakeholders. Significant heterogeneity in study designs and patient characteristics renders difficulties in establishing the most efficacious approaches. Further research is required to determine optimal strategies and the cost effectiveness of such programmes.

## INTRODUCTION

Patients undergo abdominal surgery for a wide range of indications, from a variety of benign pathology to oncological resections. This heterogeneity complicates extrapolation of surgical outcomes across patient groups and similarly, the assessment of efficacy of prehabilitation interventions to improve these outcomes. Recently defined as “*a process from diagnosis to surgery, consisting of one or more preoperative interventions of exercise, nutrition, psychological strategies and respiratory training, that aims to enhance functional capacity and physiological reserve to allow patients to withstand surgical stressors, improve postoperative outcomes, and facilitate recovery*” [1], prehabilitation has received increasing attention in both the academic and clinical literature in recent years. Effective prehabilitation requires screening for patient-related modifiable risk factors, subsequent assessment of these risk factors, and appropriate intervention.

The insult of abdominal surgery often occurs in the context of preexisting disease [e.g., gastrointestinal (GI) cancer]. Additionally, it has been shown that ~30% of patients awaiting abdominal surgery may be at risk of malnutrition preoperatively [2,3]. These factors, together with the well established association between malnutrition and adverse surgical outcomes in a variety of settings [2,4,5], makes targeted nutritional interventions a key component of the prehabilitation process for this patient cohort. This review aims to examine contemporary research over the past 2 years addressing patient selection for prehabilitation and the effects of both unimodal (nutrition alone) and multimodal (including nutrition) surgical prehabilitation, with a focus on abdominal surgery. 

**Box 1:**
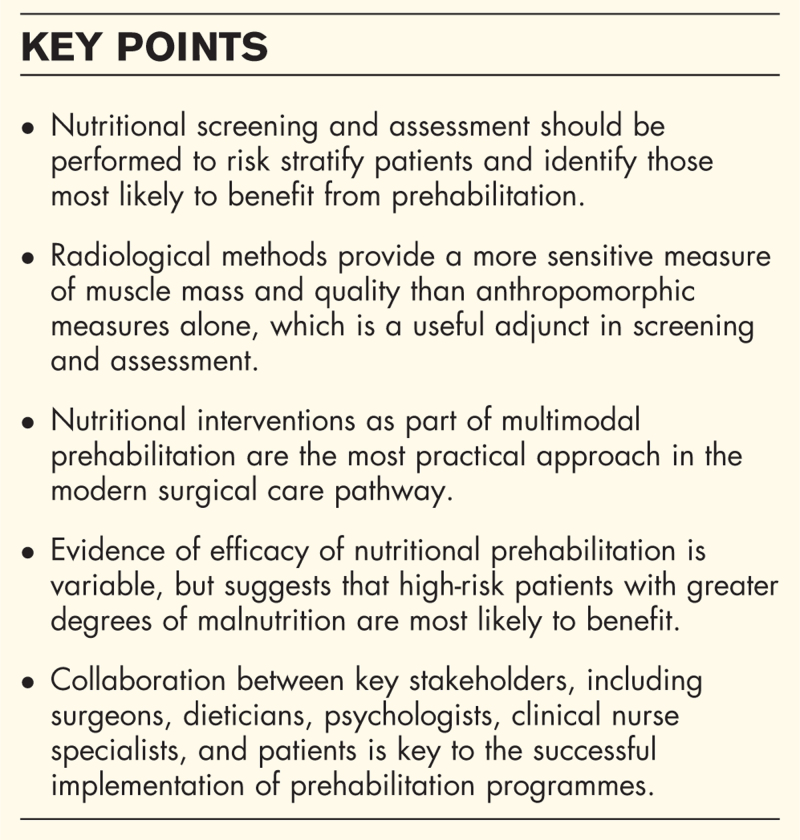
no caption available

## PATIENT SELECTION

Body composition assessments and malnutrition screening are useful tools available to risk stratify patients and identify those who are most likely to benefit from nutritional prehabilitation. Indeed, the most recent enhanced recovery after surgery (ERAS) guidelines for colorectal surgery recommend the routine use of nutrition risk screening with a validated screening tool [6]. However, to date, there remains no universally accepted consensus for the choice of tool. Recent meta-analyses have highlighted the Malnutrition Universal Screening Tool (MUST) for its ease of use, sensitivity and specificity [7,8]; although its sufficiency to justify nutritional interventions in isolation has been questioned [8].

Most nutrition screening tools, including MUST, lack identification of muscle mass and limit body composition assessment to body mass index (BMI), which may lead to missed diagnoses of malnutrition and sarcopenia – a significant limitation given the clear importance of muscle mass and function for optimal surgical outcomes [9–14,15^▪▪^]. Exemplifying the importance of muscle mass, irrespective of BMI, a large retrospective cohort study of 48 444 adults who underwent abdominal surgery demonstrated muscle mass to be inversely correlated with frailty and associated with reduced risk of 30-day hospital readmission, morbidity and mortality, independent of BMI [15^▪▪^].

Importantly, a high BMI may coexist with malnutrition and sarcopenia. As such, BMI alone may not be independently associated with postoperative risk as previously assumed, highlighting the requirement for more precise body composition assessment. A recent meta-analysis reported that although obesity conveyed an increased risk of 30-day postoperative morbidity in GI surgery [16], the same relationship was not demonstrated in subgroup analysis of lower levels of obesity (class I/II), and patients with obesity actually had a lower 30-day mortality rate. This paradoxical observation in obesity has stimulated increased interest in more precise measures of body composition which incorporate quantification of muscle mass.

For abdominal surgical patients, a promising methodology for muscle mass and quality assessment involves the use of radiological imaging modalities, such as computed tomography (CT) and magnetic resonance imaging (MRI). Utilizing this approach to determine body composition may be a more impactful risk stratification tool than BMI, as highlighted by a retrospective cohort study of 274 patients who underwent bowel resection for inflammatory bowel disease (IBD) [17^▪^]. This study categorized patients according to the presence or absence of obesity and sarcopenia, determined by CT-imaging [17^▪^]. Sarcopenic-obesity was identified as a significant independent risk factor for major postoperative complications when compared to those without sarcopenia, regardless of obesity. This effect has also been recently demonstrated in a retrospective study of 211 sarcopenic colorectal cancer patients, which concluded that sarcopenic-obesity is an independent risk factor for poor prognosis [18]. Assessment of CT-derived muscle radiodensity has received recent attention, as low muscle radiodensity has been shown to indicate myosteatosis, an indicator of poor muscle quality closely related to sarcopenia and malnutrition [19]. A single-centre cross-sectional study of 201 patients awaiting colorectal cancer surgery demonstrated superiority in stratifying those at risk of adverse outcomes using CT-derived radiodensity compared to traditional screening tools (e.g., MUST) [20]. Further, a retrospective analysis of 462 patients undergoing oesophagectomy reported higher complication and 30-day mortality rates in those with myosteatosis identified by CT [21]; a finding echoed in similar studies of gastric cancer patients [22,23]. Radiological assessment of sarcopenia using the skeletal muscle index (SMI) – skeletal muscle area (SMA) measured at the L3 vertebral level on CT scan normalized to height, has proven prognostic utility in predicting overall survival in intra-abdominal malignancy [24–26]. Given that many abdominal surgery patients will undergo diagnostic CT imaging, assessment of muscle mass/quality via this route provides a potential cost-effective, opportunistic means of patient stratification for prehabilitation. To address the need for consideration of muscle mass in nutrition screening tools, the European Society for Clinical Nutrition and Metabolism (ESPEN) has recommended utilization of the Global Leadership Initiative on Malnutrition (GLIM) criteria [27]. These include reduced muscle mass in the phenotypic domain of the diagnostic assessment.

A further area of emerging interest, especially in oncological abdominal surgery, relates to combined immune-nutritional prognostic markers such as the Prognostic Nutritional Index (PNI), the Systemic Immunoinflammatory index (SII), the CRP-albumin-lymphocyte Index (CALLY) and the Prognostic Inflammatory and Nutritional Index (PINI). These markers combine biochemical parameters related to nutritional status (e.g. albumin) with those related to immune status (e.g. monocyte and lymphocyte counts). Whilst these markers have been shown to be useful prognostic and risk stratifying tools in a variety of cancer populations [28–33], at present their role in guiding perioperative optimization is less clear than for comprehensive nutritional and body composition-based assessments. Some authors suggest these markers may identify patients who stand to benefit from nutritional and immune-modulating interventions such as protein, vitamin and mineral supplementation, or anti-inflammatory drugs [31,34]; but such recommendations are not yet backed by robust trials.

## UNIMODAL PREHABILITATION INTERVENTIONS

Considering unimodal prehabilitation, that is using nutrition support alone, oral nutrition supplements (ONS) fortified with macronutrients (proteins, fats, and/or carbohydrates) are a common strategy. Although recent primary research utilizing ONS as a unimodal intervention was not identified, a meta-analysis in those undergoing any type of elective surgery suggested that ONS provision may offer a strong protective effect against the development of surgical site infection (SSI) [35]. In contrast, in the context of only GI surgery, a Cochrane review of six randomized controlled trials reported very low certainty evidence of benefit in reducing infectious complications and low certainty evidence that standard ONS may result in little-to-no effect on noninfectious complications or hospital length of stay (LoS) compared to usual care [36^▪▪^]. There were however concerns about risk of bias in all included studies. As such, the effect of ONS on infectious complications is not clear, although analysis indicates that those with malnutrition are most likely to benefit in this regard [36^▪▪^].

The relationship between nutrition and immune function is of surgical interest due to the potential effects on wound healing, infectious complications, and postoperative morbidity. Research into the effects of immunomodulating supplements, so called “immunonutrition” (INU), has expanded in recent years with glutamine, arginine, nucleotides, omega-3 fatty acids, antioxidants (Vitamins A and C) and zinc common constituents of this approach. One double-blind randomized controlled trial (RCT) comparing an INU formulation containing arginine, nucleotides, and omega-3 fatty acids to a conventional ONS in 58 colorectal cancer patients suggested no differences in LoS nor infectious complications between the interventions [37]. However, the authors acknowledge that the small sample may not provide adequate power to detect clinically meaningful difference in these outcomes between groups. Encouragingly, an umbrella review of 16 systematic reviews and meta-analyses of perioperative INU in all GI oncological surgery reported a significant benefit on multiple clinical outcomes, including infectious complications, anastomotic leak rates, and hospital LoS [38]. These findings are however difficult to generalize as no analysis was provided for the independent effect of preoperative nutrition supplementation alone, and included studies displayed heterogeneity in intervention, population, and study design. Recent research examining the individual constituents of INU is sparse, although there has been some work exploring the impact of arginine in colorectal cancer. A nonrandomized controlled trial in this population did not show any overall benefit of preoperative arginine on surgical complication or SSI rates but did report a shorter hospital LoS [39]. Further, an RCT adding omega-3 fatty acids to arginine also reported no benefit with regards to complication rates and failed to reproduce the improvement in LoS [40]. In contrast, a meta-analysis in this population demonstrated improvements in immunological markers, such as immunoglobulins, which translated to a reduction in infective complication rates and LoS [41]. Variability in reported effects complicates the route to translate arginine supplementation into standard clinical use. The same is true for omega-3 fatty acids: although proven an important mitigator of the inflammatory process seen in cancer and after surgery, there is insufficient evidence of benefit as a preoperative intervention [42].

Overall, results from INU trials should be interpreted cautiously, as it is clear that study designs and reported outcomes suffer from high degrees of heterogeneity, making it difficult to determine overall utility as a prehabilitation strategy. The best available evidence on preoperative INU via any formulation in GI surgery has been synthesized in a Cochrane review [36^▪▪^]. Here, meta-analysis of 8 included RCTs concluded with low certainty of evidence that there is little to no difference in postoperative complications and hospital LoS. However, the authors acknowledged that none of the studies were performed in the context of ERAS, which is current clinical practice. Future research should determine efficacy within this setting via suitably powered RCT's to improve generalizability. Despite the relatively weak and contradictory evidence, the ERAS Society have recently added a strong recommendation that INU should be considered in malnourished patients with colorectal cancer [6] and ERAS Society updates for other gastrointestinal malignancies may well follow this precedent.

Finally, beyond clinical outcomes, the effect of nutritional prehabilitation, including an INU component, on quality of life (QoL) has also been recently considered in patients undergoing surgery for abdominal malignancies [43^▪^]. A feasibility study of 63 patients adopting a placebo-controlled, double-blinded, randomized design compared a protocol using protein-based ONS for 25 days, followed by an INU formulation for 5 days and a carbohydrate drink on the evening before surgery, to standard care [43^▪^]. Results of this study suggested that the nutrition intervention protected against postoperative deterioration in QoL compared to control. However, the intervention group had lower baseline demographics, likely due to higher numbers of pancreatic cancer patients, and a larger trial is required with block randomisation for type of surgery.

## MULTIMODAL PREHABILITATION INTERVENTIONS

Nutritional interventions as part of broader multimodal strategies for prehabilitation is a more active research area, with one example being the Fit4Surgery PREHAB trial. This single-centre, nonrandomized, stepped-wedge controlled trial examined the effects of multimodal prehabilitation in a variety of surgical specialties [44], with the prehabilitation including elements of exercise, nutrition, psychological support, and smoking and alcohol cessation support. Initial nutritional status was assessed using the Patient-Generated Subjective Global Assessment Short Form (PG-SGA SF) in combination with body weight and fat-free mass (FFM) assessed via bioelectrical impedance assessment (BIA). All patients received a dietician-led personalized nutritional intervention focussed on using ONS to achieve a minimum protein intake of 1.2 g/kg body weight. Initial data has been published from a cohort of 140 gynaecological cancer patients, which reports significant improvements in cardiorespiratory fitness, strength, functional exercise capacity, and malnutrition risk within the preoperative period via the prehabilitation regime [45^▪^]. However, this study is limited by missing data, lack of power calculation and relatively high baseline fitness levels within the cohort. A multicentre PREHAB RCT [46] utilized a similar assessment and intervention approach in 67 colorectal cancer patients and in a secondary analysis also reported a beneficial effect of this approach in improving nutritional status preoperatively [47].

Clinical outcomes resulting from multimodal prehabilitation in abdominal surgery have recently been addressed in a meta-analysis of 25 studies [48]. Including studies employing nutrition and exercise for at least 2 weeks preoperatively for all types of abdominal surgery, this meta-analysis reported better functional exercise capacity on a 6-min walk test (6MWT) and reduced overall complication rates in those who underwent prehabilitation. However, there were no significant differences in 90-day mortality, hospital LoS or readmission rates. Another meta-analysis, specifically examining similar prehabilitation programmes in high-risk and frail patients undergoing major abdominal surgery largely concurred [49^▪▪^]. Including 16 studies, improvements in severe postoperative complication rates and 6MWT performance was reported. Additionally, they found prehabilitation to have a favourable effect in reducing LoS, which strengthens the argument that not only is prehabilitation feasible in the presence of frailty, but that frail and/or high-risk (i.e., due to comorbidities) patients may also benefit the most from participation. In both of these syntheses, the authors acknowledged that the data is marred by high levels of heterogeneity in study protocols, reaffirming a need for better standardization of protocols to be able to firmly conclude efficacy and progress towards optimization.

## DISCUSSION AND FUTURE DIRECTIONS

Once identified, patients with malnutrition or sarcopenia may benefit most from nutritional prehabilitation. Preoperative nutritional support aims to correct malnutrition, improve resilience to the catabolic effect of surgery, attenuate the inflammatory response to surgery, and may also support the energy requirements of exercise when integrated into a multimodal prehabilitation [50]. Multimodal approaches are arguably a more pragmatic strategy which better reflect clinical views, with consensus amongst patient and professional groups that optimal prehabilitation programmes should include exercise, nutrition, and psychological support [51]. Results from available studies suggest benefit from prehabilitation in improving readiness for surgery and improving outcomes in some clinical cohorts, although further work is required to determine whether these results are generalizable to multimodal prehabilitation programmes in the entire abdominal surgery cohort and in the context of modern ERAS paradigms.

Hospital-based prehabilitation programmes may be costly and present a barrier to participation for some patients and/or healthcare organizations. As such, to try and improve compliance, models of home-based prehabilitation have been explored and shown to be a practicable alternative [52] which can achieve reductions in complication rates and hospital LoS [53]. Despite concerns about home-based programmes in high-risk patient groups, a recent study demonstrated the feasibility of their use, with high levels of adherence [54^▪^]. Technological adjuncts to support such programmes are available, including tailored apps, fitness trackers, and remote coaching and counselling. Studies addressing these methods suggest feasibility [55,56] and future studies are aiming to assess efficacy [57].

As with all interventions in a healthcare service, it is essential to consider cost effectiveness. For prehabilitation, cornerstones of this are compliance and improved clinical outcomes of reduced LoS and morbidity. Interestingly, the evidence suggests that selecting higher risk patients, such as those with frailty or undergoing more major surgery, may be more cost effective [58] and strengthens the proposition that the key to successful preoperative intervention lies in patient selection, screening, and targeted intervention. A health economic analysis is being performed alongside the ongoing PRAEP-GO trial, a multicentre two-arm parallel-group study of multimodal prehabilitation in frail elderly patients before elective surgery in Germany [59]. The economic analysis will include the 3-week preoperative period, and the subsequent 12-month follow up period. The previously mentioned Fit4Surgery PREHAB trial [44] will also publish a health economic analysis within the context of Dutch healthcare. These studies may provide high-quality evidence to support the economic value of prehabilitation, although further data will be required to extrapolate findings into healthcare settings in different countries.

Finally, restrictive timelines in oncological surgery often pose a barrier to effective implementation of prehabilitation and there is hesitance amongst some surgeons to prolong the presurgery window to allow optimization [60]. The impact of delaying such operations in this cohort has been defined as a key research priority [61].

## CONCLUSION

**FIGURE 1:**
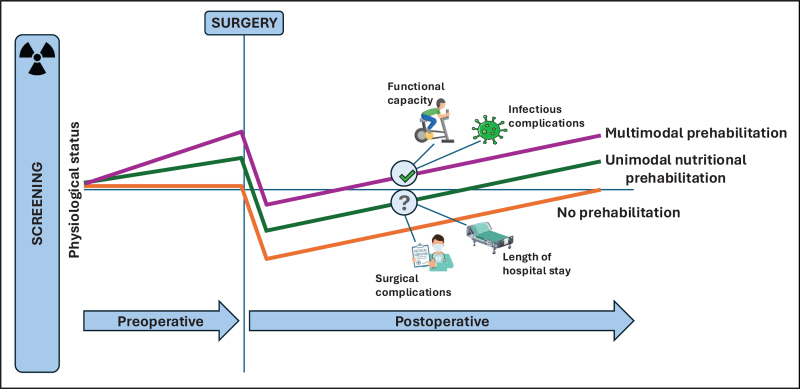
Schematic displaying the potential impact of nutritional prehabilitation on on the surgical recovery pathway.

Nutritional screening and risk stratification using validated tools can identify patients most likely to benefit from nutritional prehabilitation. Adjunctive measures, such as CT imaging, can quantify muscle quality and identify higher risk sarcopenic patients with greater sensitivity than anthropometric indices alone. Targeted nutritional interventions within a multimodal prehabilitation programme are widely considered the most pragmatic approach. Although many questions remain open for debate, it is increasingly accepted that collaboration between key stakeholders, including surgeons, dieticians, psychologists, clinical nurse specialists, and patients is key to the successful implementation of prehabilitation [62], with the overall aim of reducing the burden of the inherently challenging surgical journey (Figure 1).

## Acknowledgements

*This manuscript has been seen, reviewed and approved by all contributing authors. There are no other acknowledgements for this work.*

### Financial support and sponsorship

*This work was supported by the Medical Research Council (MR/P021220/1 and MR/X005240/1).*

### Conflicts of interest

*There are no conflicts of interest.*

## REFERENCES AND RECOMMENDED READING

Papers of particular interest, published within the annual period of review, have been highlighted as:

▪ of special interest
▪▪ of outstanding interest
